# Productivity, absence of a bull and endoparasitic nematodiosis in beef cattle farms in an upland area of East Java, Indonesia

**DOI:** 10.14202/vetworld.2020.1982-1987

**Published:** 2020-09-25

**Authors:** Widi Nugroho, Siska Aditya, Rahadi Swastomo, Aulanni’am Aulanni’am

**Affiliations:** 1 Department of Veterinary Public Health, Faculty of Veterinary Medicine, Universitas Brawijaya, Malang, East Java, 65151, Indonesia; 2 Department of Biochemistry, Faculty of Veterinary Medicine, Universitas Brawijaya, Malang, East Java, 65151, Indonesia; 3 Department of Parasitology, Faculty of Veterinary Medicine, Universitas Brawijaya, Malang, East Java, 65151, Indonesia; 4 Department of Biochemistry, Faculty of Mathematics and Natural Sciences, Universitas Brawijaya, Malang, East Java, 65145, Indonesia

**Keywords:** beef cattle farm, bull, nematodiosis, productivity

## Abstract

**Background and Aim:**

Cattle are an important economic asset for the rural community in East Java Province, Indonesia. The study aimed to provide updated data of cattle farm demography, productivity, as well as the role of the absence of a bull and nematodiosis in reduced productivity of beef cattle in an upland rural area of the province.

**Materials and Methods:**

The study was conducted in Sukowono village, Bondowoso region. A Census survey was conducted to collect data through interviews with farmers. Further, 102 fecal samples were taken systematically and processed using a double centrifugation method to investigate the endoparasitic nematodiosis in the cattle population. The demographic data, productivity, and nematodiosis were analyzed descriptively. The difference between proportions was analyzed using Chi-square with 95% confidence limit. The associations were described in risk ratio with 95% confidence interval (CI).

**Results:**

The total cattle population was 814 heads; the range of farm size was 1-7 (median: 2) cattle. Female cattle comprised 81.8% (666/814) of the cattle population but, only 5.5% (23/422) farmers kept both bull and mature female cattle. Pregnancy rate was 26.8% (145/542) of mature female cattle. The delayed first calving time appeared in 24.8% (62/250) of heifers and calving interval of >14 months occurred in 83.2% (149/179) of multiparous cows. The prevalence of endoparasitic nematodiosis was 43.1% (44/102, 95%, CI: 38.1-52.1%). Either the absence of the bull or the nematodiosis did not associate with pregnancy rate or calving interval of cows.

**Conclusion:**

This study indicates that the productivity of the cattle in the study area was low but may not associate with the absence of a bull or nematodiosis.

## Introduction

East Java Province has the largest population of beef cattle in Indonesia, with a total population of more than 4.6 million beef cattle or 27.4% of the beef cattle population across the nation [1]. The production mainly relies on a traditional farming system with very low farm sizes of <5 animals per farm [2]. Beef cattle farming in upland area of East Java was reported to be more profitable [3] and had better performance compared to those in lowland area [4]. A study reported that the average age at first mating of beef cows in an upland area of East Java was 20.4 months of age, the age at first calving was 32.4 months of age, the calving intervals were 14.5 months, and days open was 4.9 months [4]. In industrial beef production setting, among reproductive targets are, age at first mating of 12 months [5], first calving time of 24 months of age, calving interval of 12 months, weaning rate of >95% of cows, and culling rate of lower than 5% of cows [6]. In practice, some modern beef farms reach an average first mating time at 13.7 months of age [7] and a calving interval of 13 months [8] while pregnancy rate can be varied from 53% to 95% under natural service or reduced to 48-69% when artificial insemination was utilized [7]. These figures indicate that there are opportunities to improve the productivity of beef cattle farming in the upland area of East Java.

As a part of efforts to improve the productivity of beef cattle farms, the shortfall in production indicators aforementioned above and causes need to be identified and quantified so that the size of intervention and resources needed to conduct the improvement programs can be estimated. However, the information on the proportion of cows with the lower productivity and factors associated with the condition, in the context of East Java is currently not complete. A study in Lamongan East Java reported a small improvement in the reproductive performance of beef cattle by improving feeding management, but by the end of the study, the pregnancy rate was only at one-fourth of total cows [9]. This indicates that factors other than feeding play roles in low reproductive performance of cattle in the study area. The presence of a healthy bull can be useful for successful cattle breeding. Bulls have been used as a teaser to induce estrus in heifers and postpartum cows [6,10-12]. A study in upland Malang, East Java recorded that, the ratio of female to male cattle older than 1 year of age was six, almost all farmers used artificial insemination and a resulting calf crop was 58% [13]. In this considerable success of breeding; however, the role of the presence or absence of a bull in the farms remained unknown. Furthermore, helminthiasis is considered one of the major causes of economic loss in livestock production in Indonesia [14]. In East Java, few parasitic nematodes have been reported to infect cattle. A study in Jember region reported a total of 27.1% (n=314) prevalence of mixed infection with endoparasitic nematodes in cattle [15]. A study of bovine trichuriasis in Bojonegoro East Java, reported an apparently consistent prevalence of <10% in a year period [16]. Despite the identifications and prevalences, the role of these endoparasites in reduced productivity of beef cattle in East Java remains obscure.

The aim of the study, therefore, was to describe the updated demography and productivity of cattle farming in an upland area of East Java and to investigate the association of the absence of a bull and the nematodiosis, with reduced productivity of cattle in the study area.

## Materials and Methods

### Ethical approval and informed consent

The study was approved by local Government of Bondowoso Region, in the document of 070/625/430.10.5/2019. Informed consent was obtained from all participants.

### Study area

The study was conducted in Sukowono village, an upland area in the South-Eastern part of Bondowoso Region, East Java. It has an altitude of 500 asl., has an area of 3.36 km^2^, and occupied by 4985 citizens in 1650 households, mostly are engaged with agriculture as main livelihood activities [17]. The farmland, included paddy rice and horticultural farming area, is 78.5% of its total area; it is one of the most cattle populated villages in the region [17].

### Collection of farm data

A census type of data collection was undertaken on 2^nd^ and 3rd August 2019. Data were collected through interviews with household farmers using an open-ended questionnaire. For this purpose, all household farms from eight sub-villages (Indonesian language: *Dusun*) in the village were visited. As many as, eight community elders were recruited to assist eight trained enumerators during the data collection. The assistances were aimed to guide enumerators to farm locations and to communicate with the farmers who mainly speak local Madura language. Data were collected on individual cattle, including sex, age, phenotypic breeds, calving frequency of a cow, as well as on the farm structure, housing and feeding, and geographic coordinates of farms. The location of farms was described using ArcGIS 10.6 software (ESRI, Redlands, CA, USA).

### Cross-sectional survey of nematodiosis

A cross-sectional survey was conducted to comprehend the updated situation of nematodiosis in cattle in the study area and to analyze the association between the endoparasitic nematodiosis and reproductive indicators, that is, the pregnancy rate and the calving interval of multiparous cows. Data from the census were used as the population frame of the sampling survey. Fecal samples were taken systematically from seven of eight sub-villages and from one animal in every three to four farms interval at the sub-village level. At the farm level, the individual cattle included in the samples were selected as per convenience.

### Laboratory analysis

Feces were stored at 4°C boxes and immediately sent to the Tamanan Veterinary Laboratory for analysis. The laboratory was situated at a distance of half an hour from the study area. Briefly, 1 g of fecal samples was processed using a double centrifugation method with 60% sugar (w/v) as the floatation solution and examined under a light microscope with 100× [18]. All endoparasitic eggs that appeared during examination were measured using Image Raster™ (Miconos, Yogyakarta, Indonesia) for their length and width, identified based on morphology, counted, and described as the number of eggs per gram (EPG) feces.

### Statistical analysis

For the purpose of the study, female cattle were categorized into multiparous cows, primiparous cows, heifers, and female calves. A heifer was defined as female cattle at 12 months of age or older [5]. Male cattle were defined as a bull when they were older than 1.3 years of age [19]. Calving interval was calculated from cows which had calved at least twice, assuming the first calving occurred at 2 years of age, using the formula of:


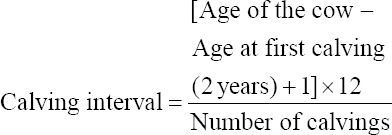


Delayed first calving time was deemed when a heifer of 3 years of age or older had not calved [4] or a heifer of 2.3-2.9 years of age which was not pregnant. Therefore, the proportion of heifers with delayed first calving time was calculated from the sum of the number of heifers of 3 years of age or older and the number of non-pregnant heifers at the group of 2.3-2.9 years of age, divide by total number of heifers:


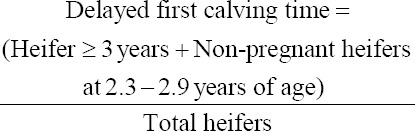


Data on prevalence were described with 95% confidence interval (CI). The difference among proportion was compared using Chi-square with 95% confidence limit. Association between the absence of bull, endoparasitic nematodiosis, and pregnancy rate or calving interval was described in risk ratio (RR), with 95% CI.

## Results

**Figure-1:**
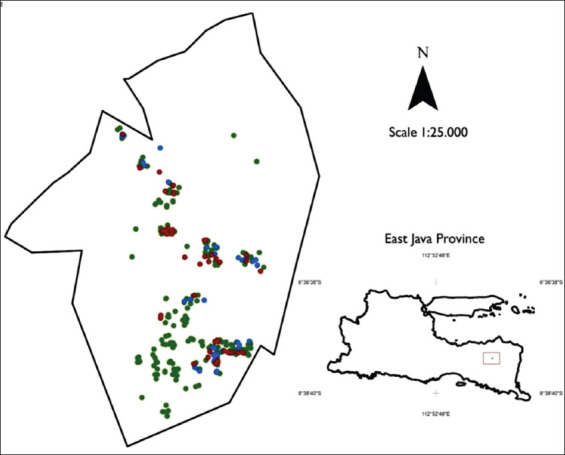
Distribution of household cattle farms in Sukowono village, Bondowoso, East Java, 2019. Colored dots indicate the farm locations, red dots indicate sample farms with the nematodiosis, blue dots indicate sample farms where the fecal test showed negative for nematodiosis. Rectangle in the map of East Java Province indicates the study area relative to the province.

The distribution of household cattle farms in the study area is shown in Figure-1. Cattle farming was practiced by 26.8% (442/1650) of households in the study area. Cattle population in the study area was 814 heads or at the average density of 3.1 cattle/Ha of the farmland area. All cattle were raised in roofed tethering. Farm sizes ranged 1-7 individuals (median: 2). The proportion of cattle with phenotypes of limousine, simmental, black cattle, and unknown mixed breeds was, respectively, 13.9% (113/814), 5.5% (45/814), 2.0% (16/814), and 78.6% (640/814) heads. The oldest mature female cattle kept by the farmer were 15 years of age, the oldest heifer was 12 years of age, and the oldest primiparous cattle were 10 years of age. The youngest pregnant female reported by farmers was at 1 year of age, while the youngest primiparous cow was at 2 years of age.

As many as 86.0% (380/442) of farmers kept mature female cattle but, only 10.0% (42/422) of farmers had bull in their farms, while only 5.5% (23/422) of farmers kept both bull and mature female cattle in the same roofed tethering. The demography of cattle population in Sukowono Village is presented in Table-1. The ratio of mature females to bulls was 12:1 (542:45). The proportion of total calves was 42.5% (124/292) of cows. Among feed sources utilized by farmers were king grass, ryegrass, rice straw, corn leaves and stalks, sugar cane leaves, as well as white tamarind leaves (*Leucaena leucocephala*).

**Table-1:** The demography of cattle population in Sukowono Village, Bondowoso, East Java, 2019.

Total population (814)					

Female (666)				Male (148)	
Cows (292)		Heifers (250)	Female calves (124)	Bulls (45)	Bull calves (103)
Multiparous (179)	Primiparous (113)	Pregnant 24.0% (60)			
Pregnant 27.4% (49)	Pregnant 31.9% (36)				

Table-2 shows that the overall pregnancy rate of cattle in the study area was low at 26.8% (145/542) of mature female cattle. A calving interval of >14 months occurred in 83.2% (149/179) of multiparous cattle. The pregnancy rate was not different among cattle in different age groups but, the proportion of cattle with a calving interval of >14 months in the group of cows of older ages was lower than that in younger cows (p=0.009). Heifers with delayed time of first calving comprised 24.8% (62/250) of heifers.

**Table-2:** Pregnancy rate, calving interval of >14 months, and endoparasitic nematodiosis in cattle in Sukowono Village, Bondowoso, East Java, 2019.

Age groups (years)	Pregnancy rate	Calving interval >14 months	Endoparasitic nematodiosis
(0-2.9)*	23.6%^a^ (54/229)	-	44.7%^a,b^ (21/47)
(3.0-6.9)	30.3%^a^ (69/228)	89.4%^a^ (93/104)	34.1%^a^ (14/41)
(≥7)	25.9%^a^ (22/85)	74.7%^b^ (56/75)	64.3%^b^ (9/14)
Total	26.8% (145/542)	83.2% (149/179)	43.1% (44/102)

* The actual range of the ages in pregnancy rate calculation is 1.0-2.9 years.

$ Calving interval was calculated as, the age of a cow minus one, divided by the number of calves; this assumes that the first calving occurs at 2 years of age.

a,b Different superscripts in the same column indicate a significant difference between proportions at the level of p<0.05

Fecal samples were taken from a total of 102 individual cattle. The ages of cattle sampled ranged from 1.5 months to 11 years, comprised 94 females and eight males. Four of the 94 females were calves. The prevalence of endoparasitic nematodiosis was high at 43.1% (CI: 38.1-52.1%). The prevalence was significantly higher in cattle at the group of 7 years of age or older, compared to younger cattle (p<0.05) (Table-2).

Three types of eggs were identified in this study: Small strongyle eggs, larger strongyle eggs, and strongyloides type of eggs. Eggs were measured and the small strongyle eggs had a length of 75.1-99.2 μm and a width of 40.0-50.5 μm, the large strongyle eggs had a length of 106.1-127.3 μm and a width of 48.6-72.3 μm while Strongyloide eggs had a length of 110.5-133.9 μm and a width of 44.9-61.3 μm.

Infection with endoparasites having small strongyle eggs was the most prevalent at 35.3% (36/102), followed by parasites having large strongyle eggs (12.7%, 13/102) and strongyloides 6.9% (7/102). The burdens, however, were low, at only a range of 1-103 EPG.

Single infections were the most common, at 32.4% (33/102). As many as 25.5% (26/102) of which was infection with endoparasites having small strongyle eggs and 3.9% (4/102) of which was infection with larger strongyle eggs whereas 2.9% (3/102) of which was infection with strongyloides eggs. Coinfection with two different endoparasites consisted of 9.8% (10/102) of infections; coinfection with larvated and larger strongyle egg parasites was 1.0% (1/102), coinfection with larvated and small strongyle egg parasites was 1.9% (2/102), while coinfection with small strongyle and larger strongyle egg parasites was 6.9% (7/102). Coinfection with three different endoparasites was least common, only at a prevalence of 1.0% (1/102).

Nematodiosis in group of ≥7 years of ages was higher than that in younger cattle (p<0.05), but there was no differences in the prevalence of nematodiosis among sex or breeds (p>0.05). Further, statistical analyses indicated that there was no association between the absence of a bull or the nematodiosis and pregnancy rate or calving interval in different groups of ages (Table-3).

**Table-3:** The absence of bull, nematodiosis, and their association with reproductive indicators of cows in Sukowono village, Bondowoso, East Java, 2019.

Absence of bull	Reproductive indicators		Risk ratio (CI)

	Pregnancy status		

	Not pregnant	Pregnant	
Absent	374	136	RR: 1.0 (CI: 0.8-1.3)
Present	23	9	

**Calving interval (months)**			

	>14	≤14	
Absent	138	29	RR: 0.9 (CI: 0.7-1.1)
Present	11	1	

**Endoparasitic nematodiosis**	**Reproductive indicators**		**Risk ratio (CI)**

	**Pregnancy status**		

	**Not pregnant**	**Pregnant**	

Infected	28	9	RR: 1.0 (CI: 0.8-1.3)
Not infected	39	14	

**Calving Interval (months)**			

	>14	≤14	RR: 0.8 (CI: 0.6-1.1)
Infected	10	3	
Not infected	16	1	

RR=Risk ratio, CI=Confidence interval

## Discussion

This study provides an updated baseline data of traditional cattle farming in an upland area of East Java. The study, however, based the data from the interview with farmers which relied on recalling of the respondents. Thus, one should be careful in interpreting the result of the study due to potential recalling bias. This study indicates that cattle farming was an important part of livelihood activities for many households in the study area. The high proportion of households who kept mature female cattle suggests that the main purpose of the farming was for breeding.

The majority of farmers in the study area may keep cattle for breeding, but current study showed that there were few old heifers which had never calved or had a very long calving interval, which remained in the farms. These imply that besides breeding, some farmers might keep cattle for other reason. It was reported previously that some villagers in East Java kept cattle for social status, where number of cattle owned was more important than its economic value *per se* [20]. However, the farming purpose of having a larger number of cattle can actually be achieved by successful breeding; therefore, these proportion of farmers needs to be trained on ways to improve reproductive performance of their cattle.

Reproductive performance of cows in the study area was very low, indicated by low pregnancy rate, high proportion of cows with longer calving interval, and high proportion of heifers with delayed first calving time. However, the reduced pregnancy rate or longer calving interval did not seem to associate with the absence of a bull in a farm. Bulls were kept by only a small proportion of farmers in the study area. It means that the majority of these farms relied heavily on accurate estrus observation by farmers and timely available bull for a service or semen for artificial insemination. Failure to comply with either factor would reflect into increased length of calving interval or delayed first calving time. Therefore, whether practices of estrus observation and insemination services in the study area contribute to low productivity of cows, warrant further research.

On the other hand, the calf crops in current study, estimated by the ratio of calves to cows, were one half higher than the pregnancy rate. The higher calf crop compared with pregnancy rate in the current study may reflect a longer calving interval. In addition, the calf crop in the current study was slightly lower than that estimated from artificial insemination recording of cows in Bondowoso region, which reported around half of the number of cows [21]. It indicates that the calf crop in the study area, was not superior in the region.

The prevalence of nematodiosis was high in the current study,, but the level of nematodiosis was mild, expressed by low number of EPG feces [22]. Mild infection despite a high prevalence might explain why nematodiosis was not associated with pregnancy rate or calving interval in different groups of age, even in the group of older cows where the prevalence was higher. These findings also lead to further speculation of resistance of the cattle in the study area against parasitism. Cattle infertility was reported to link with heavy parasitism [23,24], but resistance of certain breeds of cattle against parasitic infections have been reported [25,26]. In addition, the lack of the role of nematodiosis in reduced productivity of cows in the current study suggests that the importance of nematodiosis in livestock production among regions in Indonesia could be different; thus, cannot be generalized.

## Conclusion

This study indicates that cattle farming in the study area was intended mainly for breeding, low in productivity, but the absence of bull or the nematodiosis did not associate with low productivity. The causes of low productivity of beef cattle farms in the study area remain an open question and await investigation.

## Authors’ Contributions

WN: Conceptualization, Methodology, Validation, Formal analysis, Investigation, Resources, Data Curation, Writing – Original Draft, Writing – Review and Editing, Visualization, Supervision, Project administration. SA: Conceptualization, Methodology, Validation, Writing – Review and Editing. RS: Investigation, Resources, Data Curation, Supervision. AA: Conceptualization, Methodology, Validation, Resources, Writing – Review and Editing, Supervision, Project administration, Funding acquisition. All authors have read and approved the final manuscript.
